# The Effects of Age and Height on Gait Smoothness in Adolescent Athletes †

**DOI:** 10.3390/children11020223

**Published:** 2024-02-09

**Authors:** Lindsay M. Clarke, Resa M. Jones, Shivayogi V. Hiremath, Corinna Franklin, W. Geoffrey Wright, Carole A. Tucker

**Affiliations:** 1 Department of Health and Rehabilitation Sciences, College of Public Health, Temple University, Philadelphia, PA 19122, USA; shiv.hiremath@temple.edu (S.V.H.); william.geoffrey.wright@temple.edu (W.G.W.); 2 Department of Epidemiology and Biostatistics, College of Public Health, Temple University, Philadelphia, PA 19122, USA; resa.jones@temple.edu; 3 Fox Chase Cancer Center, Temple University Health, Philadelphia, PA 19140, USA; 4 Department of Pediatric Orthopedic Surgery, Yale University School of Medicine, New Haven, CT 06520, USA; corinna.franklin@yale.edu; 5 Department of Nutrition, Metabolism, and Rehabilitation Sciences, School of Health Professions, University of Texas Medical Branch, Galveston, TX 77555, USA; cartucke@utmb.edu

**Keywords:** adolescent, sports injury, gait, harmonic ratio, adolescent motor awkwardness

## Abstract

(1) Background: Despite evidence of increased rates of sports injury during the years surrounding peak growth in adolescents, little is known regarding the relationship between adolescent growth and gait stability. The aim of this study was to gain a better understanding of how chronological age and height relate to gait stability in both male and female adolescents. (2) Methods: Participants (N = 67; females: *n* = 34, ages 8.7–15.9 years; males: *n* = 33, ages 10.0–16.7 years) completed two trials of treadmill walking at varying speeds: the preferred walking speed and 30% above and below. Trials were separated by a bout of fatiguing exercises. HarmonicRatios of the trunk, calculated from acceleration signals taken during walking, were used to quantify gait stability. Data were separated by sex and relationships between height and chronological age, and HarmonicRatios were assessed using multiple linear regression. (3) Results: Females’ HarmonicRatios improved with chronological age both before and after fatigue. Males’ HarmonicRatios increased with chronological age before fatigue; however, this effect was eliminated post-fatigue. Females’ height was negatively associated with HarmonicRatios post-fatigue. Males’ height was positively associated with HarmonicRatios pre-fatigue. (4) Conclusions: The study findings suggest sex differences in the effects of fatigue on gait stability during adolescence. In both sexes, HarmonicRatios increased with chronological age. These improvements were eliminated for males and altered for females with fatigue. The results of this study indicate the need for the reevaluation of sports progression based on chronological age in adolescents.

## 1. Introduction

A stable gait can be defined as a gait “that does not lead to falls” [1]. The ability to produce a stable gait both is an essential component of navigating tasks of everyday life and forms the basis upon which many sport-specific motions are built. Some kinematics of gait appear to achieve an adult-like pattern by 7–8 years of age [2]; however, gait parameters including step length [3], cadence [3], single and double limb support time [3], and smoothness of trunk motion [4] have been shown to continue to change in adolescents. Alterations to these gait parameters, along with a number of others, have been linked to increases in fall risk for older adults [4]; however, investigations into the timing and impact of changes to these parameters on adolescents are sparse. The knowledge gap created by the relative lack of research is further complicated by the potential for differential development of gait parameters due to sex-based variations in physiological and physical development [5]. A primary difference that may impact gait development is the timing of growth relative to chronological age between the sexes. On average, females reach their peak height velocity (0.083 m/year), the highest rate of growth during the adolescent growth phase, at 11.5 years of age [5]. Males, however, do not reach their peak height velocity (0.095 m/year) until an average of 13.5 years of age [5]. Changes to the nature of sports participation have been traditionally based largely on chronological age, irrespective of sex [6]. The difference in relative growth timing may have direct effects on sport-related injury risk due to increases in the intensity, volume, and speed of sport practice and gameplay. Adolescent athletes experience an increase in the incidence of sports injuries of a traumatic nature during the year of peak height growth compared to the years prior [7]. This increase in the prevalence of traumatic injury is chronologically followed by an increase in the incidence of overuse injury in the years directly following the year of peak height growth [7]. The increase in the occurrence of injury during peak growth and the subsequent shift in injury type from traumatic to overuse between the growth year and post-growth year suggests an alteration to movement patterns around the time of physical growth. The alteration to movement patterns may first create an unbalanced or unstable movement system, resulting in an increase in traumatic injuries, followed by compensation strategies for the altered mechanics, resulting in an increase in overuse injuries. A better understanding of the interplay between physical growth, changes in movement dynamics, and changes in injury risk throughout the period of adolescent development is a crucial step toward reducing the prevalence of sports-related injuries in this population.

Adolescent motor awkwardness (AMA) has been loosely defined as a temporary decline in an individual’s motor control during the years surrounding the adolescent phase of growth [8]. Though AMA is frequently discussed anecdotally by parents, coaches, therapists, and clinicians, the specifics of the phenomenon have yet to be thoroughly scientifically characterized or explored. A better understanding of the timing, underlying mechanisms, and overall impacts of AMA on youth athletes is crucial to their proper development in an increasingly intense sport environment. During the adolescent growth phase, the size of individual body segments changes both absolutely and relative to each other. This change has the potential to impact the efficacy and efficiency of previously employed stabilizing strategies during gait [7,9,10,11] as the mechanics of controlling each segment and the forces needed to control each segment change. Additionally, changes to absolute segment length precede muscle lengthening. This temporarily alters the muscle mechanics and torque production capabilities of various body segments and, thus, the ability to maneuver in a coordinated manner [12]. Many aspects of the progression of adolescent sport difficulty, speed, and the intensity of both practice and gameplay are based primarily on chronological age [6] but do not account for variations in the timing of physical manifestations of AMA. As coaches and trainers are typically unable to test for the biological or developmental age of their athletes, understanding the progression of gait stability relative to chronological age during adolescence may be helpful in refining training strategies for subgroups within a wider age range. To our knowledge, while the development and maturation of gait in children have been extensively studied, there is little to no evidence to date regarding the timeline of changes to gait stability specifically in adolescents. This study aims to begin to understand the timeline of changes to gait stability, which may result as a physical manifestation of AMA. The expansion of our knowledge regarding changes to gait stability in adolescents, particularly through testing both the un-fatigued and fatigued state, could have long-term benefits in youth athlete development.

The goals of this study were to (1) better characterize cross-sectional differences in gait stability in individuals across the span of ages surrounding the national averages for peak height velocity (i.e., males of 10–17 years of age and females of 8–15 years of age), specifically investigating the smoothness of trunk motion measured as a harmonic ratio (HarmonicRatio), and (2) identify potential sex differences in the associations of height and chronological age with gait stability during these same age ranges. We hypothesized that HarmonicRatios would increase with age in both males and females but that there would be differences between the sexes in how the increase occurs. In regard to the relationship between height and HarmonicRatios, we hypothesized that taller individuals would display lower HarmonicRatio values when correcting for chronological age.

## 2. Materials and Methods

### 2.1. Subjects

Participants for this study were a convenience sample of youth athletes from the greater Philadelphia area (N = 67; *n* = 33 males, *n* = 34 females). Athletes were recruited through local sports teams, businesses, and summer camps. The participants were included in the study regardless of the sport in which they participated. However, athletes were only enrolled in the study if they were, at the time of their participation in the study, involved in a competitive sport at least three times per week.

The exclusion criteria were any lower-extremity musculoskeletal injury, other injury that affected gait, or the presence of a neuromuscular condition within the past six months.

Parental consent and informed child assent were obtained before participation in this study, as approved by the Institutional Review Board of Temple University (IRB #26366).

### 2.2. Protocol Overview

**Figure 1:**
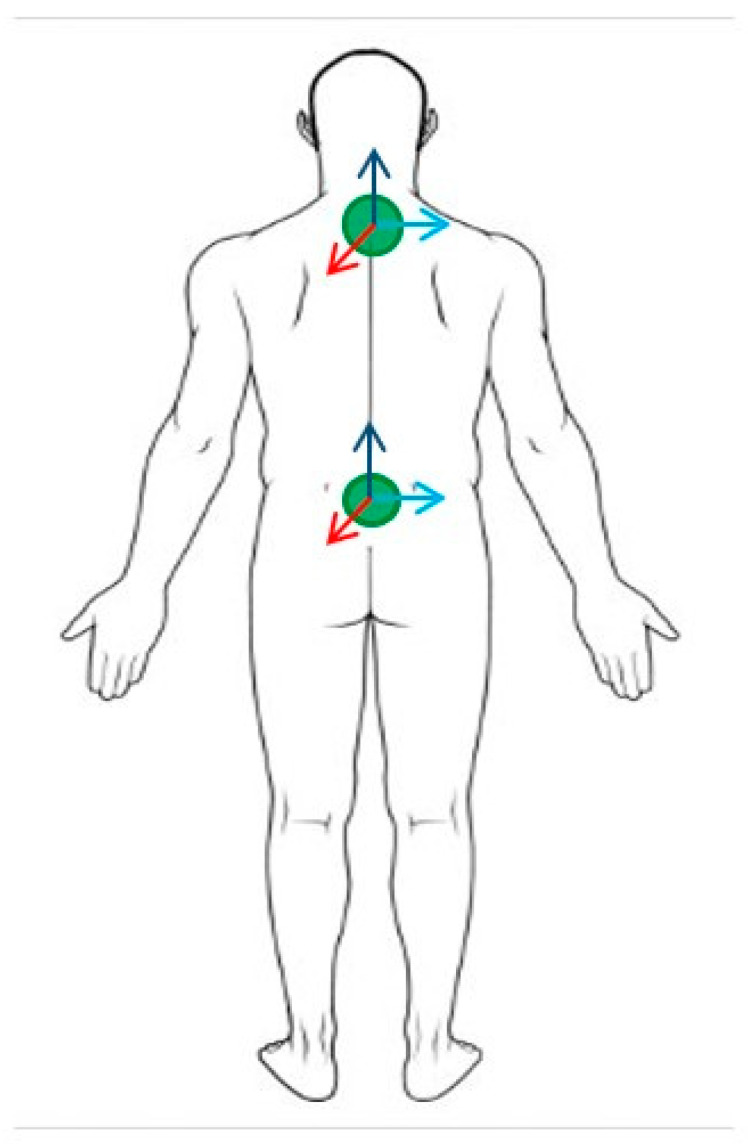
Placement of IMU sensors on each participant. Arrows indicate planes of acceleration signals recorded (i.e., ML, AP, and V).

The participants executed a standardized series of tasks in a single 2-h lab-based testing session at Temple University. The primary physical and demographic characteristics recorded were chronological age and standing height. Standing height was measured for each athlete and recorded using a standard procedure and tape measure. Chronological age was self-reported and recorded in years and months. Trigno inertial measurement unit (IMU) sensors (Delsys, Natick, MA, USA) were placed (Figure 1) on the upper trunk at the level of the seventh cervical vertebra (C7) and on the lower trunk at the level of the fifth lumbar vertebra (L5). Sensors were also placed on the right and left recti femoris for within-test observational purposes to ensure the efficacy of the fatigue protocol in engaging the muscles of the leg. The location of placement for each sensor was determined through manual palpation of the areas indicated in green in Figure 1. The sensors were mounted superficially to the skin with double-sided adhesives at the determined locations. Following sensor placement, the participants completed a researcher-directed 15-min warm-up on the treadmill to familiarize themselves with the treadmill environment. During this warm-up period, the participants began at a researcher-selected slow walking pace of 0.8 m/s. At the culmination of each subsequent 45-s interval, the speed of the treadmill was increased in increments of 0.2 m per second until the subject reached their walk-to-run transition speed. The participants were given 45 s at each speed interval to ensure that they were able to reach their natural gait pattern at that speed. As the walk-to-run transition approached, many initially began to run as the speed increased and then settled back into a fast walking gait within a few strides. Once the participant remained in a running gait for the entirety of the interval, the treadmill speed was increased one increment further. Following this brief bout of running gait, the speed of the treadmill was decreased in the same sequential increments at the same time intervals. At each descending speed, once a full interval of walking gait was resumed, the participants were asked to characterize how their current walking speed felt relative to their preferred comfortable walking pace (i.e., faster, slower, or right on). The participants were instructed to evaluate their current speed relative to the speed at which they would walk down the street if they were not in a rush. Slow (SWS) and fast (FWS) walking speeds were calculated, respectively, to be 70% and 130% of a participant’s self-reported preferred walking speed (PWS) [13]. In two cases, the calculated fast walking speed fell at the participants’ walk-to-run transition speed. In these cases, the fast walking speed was adjusted by 0.2 m/s to be below the participant’s walk-to-run transition speed. Paced walking above the natural walk-to-run transition speed has been shown to significantly increase attentional and cognitive demand [14], which, in turn, impacts gait performance.

After the warm-up, the participants were given a period of no more than 4 min to rest before beginning the first of two 9-min walking trials on the treadmill. Each trial consisted of three consecutive 3-min segments: the first segment at SWS, followed by a segment at PWS, and then finally a segment at FWS. Accelerations were recorded from the sensors placed at the upper and lower trunk in the anterior–posterior (AP), medio-lateral (ML), and vertical (V) planes of motion at a sampling rate of 148 samples/s during both walking trials.

### 2.3. Muscular Fatiguing Protocol

Following the first 9-min treadmill walking trial, the participants completed a fatiguing exercise protocol consisting of 10 min [15] of leg-focused movements. At the beginning of each of the 10 min, the participants were asked to perform either 20 squat jumps or 20 lunges, either reverse lunges or forward lunges. The exercise type alternated each minute for a total of five sets of each. The remaining time in the minute following the completion of the designated number of repetitions of the exercise served as the participants’ rest period. This fatiguing protocol was selected for its ecological validity. The structure of the protocol closely mimics the activities of regular sports practice, therefore creating fatigue patterns similar to those that athletes are expected to endure during their sport activities [16]. The subjects were monitored during the totality of the exercise protocol to ensure both the proper execution of each movement pattern and the completion of the entire set of repetitions. Upon completion of the fatiguing protocol, the athletes were instructed to rest for a period of five minutes prior to the initiation of the second walking trial. This rest period served as a window to allow their heart rates to return to their resting values and for the spatiotemporal parameters of gait to return to the baseline [15].

### 2.4. Dependent Variable Theory and Calculation: HarmonicRatio

The HarmonicRatio is the ratio between the powers of the in-phase, or symmetric with the base harmonic of the signal, to those of the out-of-phase, or not symmetric with the base harmonic, components of a cyclical signal. For this study, the HarmonicRatio was used to quantify the smoothness of trunk motion in the three primary planes of motion (i.e., AP, ML, and V) at both the upper and lower trunk. A higher HarmonicRatio value indicates a greater power of the in-phase components of the signal compared to the out-of-phase components. In human movement analysis, this is indicative of a greater rhythmicity or smoothness of motion. A lower HarmonicRatio represents a higher power of the out-of-phase components compared to the in-phase components, indicating a lower rhythmicity or smoothness of motion [13,17].

The HarmonicRatio has been previously validated and used in human movement analysis, primarily as a measure of gait smoothness and stability [17,18,19]. Additionally, the HarmonicRatio has been shown to reflect subclinical alterations in gait in older healthy adults [20], individuals with neuromuscular diseases that have not yet impacted day-to-day functioning of gait [21,22], and individuals with known balance deficits [23]. Due to the fact that the target subject pool for this study was healthy individuals with a relatively unimpaired gait (i.e., the participants had no clinical neurological conditions or gait-altering conditions), some of the traditionally analyzed spatiotemporal measures of gait may not have been sensitive enough to detect alterations to gait between those who were growing and those who were not [24]. In addition, many spatiotemporal measures used for gait analysis may not specifically identify alterations to trunk motion. The smoothness of trunk motion is a key feature of stable gait, as it forms the basis for the stability of the head and, therefore, both the visual and vestibular balance systems. Erratic trunk motion can impair gait and increase fall-related injury risk through changes in the ability to stabilize the head. Research using methods that detect subtle alterations to gait stability is necessary to determine the effects of physical growth on gait, which may not yet meet the criteria for clinical coordination disruption but still present an increase in the risk of fall and injury [5,6].

**Figure 2:**
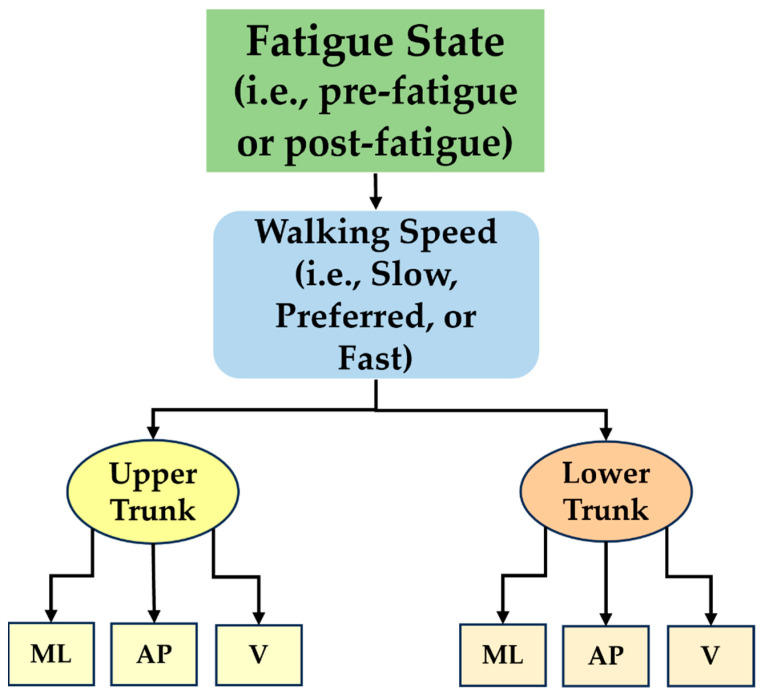
Flowchart depicting the breakdown of the HarmonicRatio variable.

To obtain HarmonicRatios, acceleration signals in the ML, AP, and V planes of motion were taken from the surface-mounted Trigno sensors (Delsys, Natick, MA, USA) located at both the upper and lower trunk. This resulted in six distinct values for HarmonicRatios for each walking speed and in each walking trial: three for the upper trunk (HarmonicRatio_ML_, HarmonicRatio_AP_, and HarmonicRatio_V_) and three for the lower trunk (HarmonicRatio_ML_, HarmonicRatio_AP_, and HarmonicRatio_V_). The middle minute of each of the three three-minute gait speed segments within each walking trial was used for analysis. A full minute of walking was used to ensure the inclusion of enough strides to achieve an accurate HarmonicRatio calculation [25]. The use of only the middle minute of each segment also limited the potential inclusion of strides around the speed transition. The mechanics of these transitional strides may have been impacted by either the actual change in walking speeds or by the anticipation of a transition to the next speed. Each selected segment was passed through a 4th-order Butterworth filter and converted into a frequency domain using a fast Fourier transform, which was used to identify the harmonics of the signal’s fundamental frequency. As HarmonicRatios must be calculated within each stride [13,25], strides were delineated based on signal peaks and then split into individual strides prior to harmonics calculations. The power of the first 20 harmonics from the processed signal was used in calculating the HarmonicRatio for that segment of data [13,18,25]. During gait, the signals produced in the AP and V directions are biphasic, having two peaks per stride. The signal resulting from motion in the ML direction, however, is monophasic, with only one peak per stride. Therefore, the even harmonics of the signal represent the in-phase components in AP and V signals, and the HarmonicRatio is calculated as the ratio of the powers of the even to the odd harmonics. Due to its monophasic nature, the odd harmonics of the ML signal represent the in-phase components, and the HarmonicRatio calculation is reversed from that of the AP and V signals [18]. The resulting HarmonicRatios calculated from each individual stride within the selected minute were averaged to produce an overall HarmonicRatio for each gait speed, sensor location, plane of motion, and fatigue status combination. This resulted in 36 total unique values for HarmonicRatio, 18 pre-fatigue and 18 post-fatigue, for each participant (Figure 2).

### 2.5. Independent Variables and Covariates

Sex was assessed as a binary variable based on participants’ self-reported responses. Two primary independent variables were identified: (1) chronological age (in years and months) and (2) height in meters. During adolescence, particularly in the years surrounding the peak height velocity, substantial physical changes occur over a period of months. Therefore, the traditional model of analyzing age in discreet epochs, rather than as a continuous variable, may result in the exclusion of some of the more minute changes occurring over this time frame. To maximize the ability to accurately characterize the changes seen across the age ranges and body sizes, both chronological age and height were analyzed as continuous variables.

Athletes’ self-reported primary sport was recorded and categorized as gait-based (e.g., basketball, baseball, and soccer), semi-gait-based (e.g., gymnastics and dance), and non-gait-based (e.g., rowing and equestrianism). The sport type was assessed as a covariate. The participants’ absolute gait speed, as opposed to the gait speed relative to their individual preferred speed, was also recorded and assessed as a covariate.

## 3. Statistical Analysis

All data were stratified into self-reported male and female sex groups for analysis and tested for normality using a Shapiro–Wilk test. Descriptive statistics, including averages of age, height, and walking speeds, were calculated for each sex group. These calculated sex group means were compared using independent-sample t-tests. HarmonicRatios were calculated as described previously, utilizing a custom Matlab (Version 2020-Math Works, Inc., Natick, Ma, USA) code. Multiple linear regression modeling was conducted using RStudio (RStudio Team 2020) to assess the relationships of age and height with each unique HarmonicRatio pre- and post-fatigue stratified by sex.

The final stratified multiple-linear-regression models for each unique HarmonicRatio at pre- and post-fatigue were fully adjusted for both age and height. Gait speed and sport type were not included in the final model, as they were found to have no significant impact. The alpha level for all analyses was set to 0.05. Analyses were adjusted using Holm–Bonferroni family-wise error correction.

## 4. Results

### 4.1. Demographics

Table 1 depicts the results of the descriptive statistics and group mean comparisons. The mean height for males was 1.66 m (range = 1.33–1.88) with a mean age of 13.46 years (range = 10–16). Females, on average, were both shorter and younger than males with a mean height of 1.57 m (range = 1.39–1.76) and a mean age of 12.13 years (range = 8–15). The mean age, height, walk-to-run transition speed, and means of both the preferred and fast walking speeds significantly differed between the sex groups (Table 1). Male participants were, on average, taller and older than female participants and transitioned from walking to running at a higher speed. Males also indicated a higher preferred walking speed compared to females.

Table 2 and Table 3 provide the results obtained from the adjusted linear regression models for each sex group, respectively, assessing the association between the participants’ chronological age and HarmonicRatios adjusted for height (Table 2) and between the participants’ height and HarmonicRatios adjusted for chronological age (Table 3).

### 4.2. Group Results: Females

After adjusting for height, females exhibited significant relationships between HarmonicRatios and chronological age in several plane-of-motion, walking-speed, and fatigue-state combinations. HarmonicRatio_AP_ of the upper trunk showed a significant relationship with chronological age at all walking speeds in the pre-fatigue state (slow walking speed: β = 0.158 ± 0.055, *p* = 0.007; preferred walking speed: β = 0.249 ± 0.084, *p* = 0.005; fast walking speed: β = 0.275 ± 0.083, *p* = 0.002). Post-fatigue, chronological age was found to have a significant positive relationship with HarmonicRatio_AP_ at the upper trunk during fast walking (β = 0.318 ± 0.095, *p* = 0.002) and with HarmonicRatio_V_ at the upper trunk during both slow walking (β = 0.166 ± 0.045, *p* = 0.001) and preferred walking speed (β = 0.173 ± 0.049, *p* < 0.001). There was no linear relationship found between chronological age and HarmonicRatio_ML_ at any speed or in either fatigue condition at the upper trunk. In females, chronological age was not found to have a significant relationship with the HarmonicRatio at the lower trunk in any plane of motion, at any gait speed, or in any fatigue condition following adjustment for height.

Following adjustment for chronological age, females also exhibited significant negative relationships between the HarmonicRatio and height in several conditions. Post-fatigue, HarmonicRatio_V_ of the upper trunk during both slow walking (β = −0.098 ± 0.027, *p* = 0.001) and at the preferred walking speed (β = −0.092 ± 0.025, *p* = 0.001) both showed a significant negative relationship with height. HarmonicRatio_AP_ of the upper trunk during post-fatigue fast walking (β = −0.144 ± 0.052, *p* = 0.009) also showed a significant negative relationship with height. After adjusting for chronological age, the HarmonicRatios of the lower trunk were not associated with height any plane of motion, speed, or fatigue state.

### 4.3. Males

After adjusting for height, males’ chronological age showed a significant positive relationship with HarmonicRatio in three instances, all of which were in the pre-fatigue state. HarmonicRatio_ML_ at the lower trunk (β = 0.313 ± 0.109; *p* = 0.007) showed a significant positive relationship with age during fast walking, as did HarmonicRatio_AP_ at the upper trunk (β = 0.244 ± 0.097; *p* = 0.017) and HarmonicRatio_V_ at the upper trunk (β = 0.356 ± 0.083; *p* < 0.01). No other relationship between age and HarmonicRatio was found to be significant after adjusting for height.

After adjusting for chronological age, height exhibited a significant negative relationship with HarmonicRatio_V_ at the upper trunk during fast walking in the pre-fatigue state (β = −0.078 ± 0.029; *p* = 0.02). The relationship between height and the HarmonicRatio was not found to be significant for any other condition after adjusting for chronological age.

## 5. Discussion

The objective of this study was to investigate factors possibly affecting the stability of walking gait in adolescents during the years surrounding peak height velocity. Specifically, we aimed to better understand sex group-related differences in the relationships between age, height, and smoothness of trunk motion during walking gait. We initially hypothesized that males and females would differ in how the dynamic stability of their trunk motion during gait (i.e., HarmonicRatio of the trunk) would change with age in the years surrounding peak height growth. We also hypothesized that taller individuals would be less stable than shorter ones when accounting for age, regardless of sex group.

The overall findings of this study were that females’ HarmonicRatios improved with chronological age both before and after fatigue. In males, HarmonicRatios increased with chronological age before fatigue; however, this effect was eliminated post-fatigue. In females, height was negatively associated with HarmonicRatios post-fatigue, while in males, height was positively associated with HarmonicRatios pre-fatigue. Height was not significantly associated with HarmonicRatio in any other condition.

To our knowledge, no previous study has specifically investigated the relationship between chronological age and harmonic ratio during adolescence. Therefore, no studies exist for a direct comparison with the results presented here. Further research on this specific topic is crucial to creating a better understanding of AMA and of the evolution of gait and gait stability throughout adolescence.

### 5.1. Trunk Motion and Age

Prior to undergoing a fatiguing bout of exercise, older females demonstrated a clear improvement in HarmonicRatio_AP_ at the upper trunk over younger females at all tested walking speeds. As a higher HarmonicRatio is indicative of a smoother and more stable gait [17], this finding led us to believe that the motor control of the trunk in the AP direction in females may continue to develop throughout the time frame measured. This progression in smoothness of motion may be due to a number of physical or social factors and requires further investigation. In females, following fatiguing exercise, the increase of the HarmonicRatioAP of the upper trunk with increasing age was limited to only the fast walking speed. Interestingly, following fatiguing exercises, the remaining positive relationships between age and the HarmonicRatio of the upper trunk switched during slow and preferred walking from HarmonicRatio_AP_ to HarmonicRatio_V_, potentially indicating a change in trunk stabilization strategies in adolescent females from pre- to post-fatigue. Further studies and analyses are needed to determine the mechanism of this change.

In males, age-related improvements to HarmonicRatios were noted only during fast walking, with older males showing higher HarmonicRatio indicative of greater stability during fast walking compared to younger males [17]. Chronological age, however, showed no significant effect in males on HarmonicRatio during slow walking or at their preferred walking speed. Improvements to HarmonicRatios with increasing chronological age were noted in male participants pre-fatigue but not following the fatiguing bout of exercise. These results suggest that older males may have already developed the muscular control strategies to stabilize their gait during fast walking that have not yet been developed in younger males but that those strategies fail once they become fatigued. For male athletes in the middle and high school age range, this means that, though chronologically older males may exhibit more stability than younger males at the start of a practice or game, this may not hold true as the practice or game progresses and they become fatigued. Thus, the practice of determining the timing of increases in training and exercise loads and intensities primarily on chronological age in middle school and high school sports should be re-evaluated.

Observationally, following the fatiguing bout of exercise, a substantial subset of the participants in this study displayed a noticeable decrease in activation of the recti femoris at slow walking and at their preferred walking speed compared to their pre-fatigue walking. However, once they progressed to the fast walking segment, these participants re-engaged the recti femoris. This change in muscle activation patterns from slow to fast walking post-fatigue may have played a role in the pre-to-post-fatigue changes seen in females and differences seen in the relationships between age and HarmonicRatios from pre-to-post-fatigue in females. Pre-fatigue, the significant relationship between HarmonicRatios and chronological age in females was seen at the upper trunk in the AP direction at all walking speeds. The same was true post-fatigue for the fast walking speed; however, the significant relationship switched from the AP to the V direction at both the slow and preferred walking speeds. As the fast walking speed post-fatigue coincided with the activation of the recti femoris more similarly to that seen during pre-fatigue walking, the shift in plane may have been the result of changes in muscle activity. More studies targeted at understanding the activation patterns of various lower-extremity muscle groups during walking in adolescents, and sex differences therein, are needed to fully understand this phenomenon. As no relationship was found in this study between chronological age and HarmonicRatio_ML_ of the upper trunk in either sex, it appears that the lateral control of the trunk during gait may have already reached a mature pattern by the age of physical growth.

The between-sex differences in pre-to-post-fatigue relationships between HarmonicRatio and chronological age are of note: males’ relationship between age and HarmonicRatios was eliminated in the fatigued state, while in females, the relationship between chronological age and HarmonicRatios shifted the plane of motion with fatigue. In adolescents, the timeline of muscular development relative to bone lengthening differs between male and female individuals. This difference in growth patterns may create distinct effects of fatigue on muscle activation and motor control between the sexes. Second, male and female morphology diverges during this age range and period of development [10], potentially necessitating the evolution of different movement patterns, control strategies, and muscle activation patterns.

On average, the male and female groups differed in the number of years they were removed from the national average age at which their sex group reached peak height velocity (i.e., the female group was an average of 1.1 years older than their sex group’s average age at peak height velocity, while the males were only an average of 0.4 years older than their sex group’s average age at peak height velocity) [5]. As many aspects of development occur rapidly during this time frame, this seemingly small disparity in average relative age may in fact play a significant role in the between-sex differences seen in the relationships between the HarmonicRatio and chronological age. This may indicate that gait stability progresses on a timeline more similar to biological or developmental age than chronological age. Further research is needed to better define the timeline of disturbances to motor coordination and gait relative to chronological and developmental age.

Though our initial hypothesis that HarmonicRatios would increase as chronological age increased was upheld for some configurations of the HarmonicRatio, in many instances, no significant relationship was shown between the two variables. Previous research has shown that various spatiotemporal measures of gait reach their mature patterns at different chronological ages throughout the age range included in this study [3]. The lack of a linear relationship between the HarmonicRatio and chronological age for some configurations may indicate that motion in these directions or at these relative gait speeds may have reached full stability prior to the ages included in this study. Alternatively, the lack of a linear relationship may indicate a non-linear relationship of improvement across this age range. The presence of non-linear maturation of gait stability during adolescence is a potential explanation that requires further, more longitudinal investigation to further clarify the timeline of development.

### 5.2. Trunk Motion and Height

In the female group, height was found to be a significant predictor of HarmonicRatios following a bout of fatiguing exercise at HarmonicRatio_V_ at the upper trunk during both slow and preferred walking and of HarmonicRatio_AP_ at the upper trunk at the fast walking speed. Additionally, the relationships between height and HarmonicRatios in two more post-fatigue conditions trended toward significance, though they did not reach it following adjustment for age. This indicates that taller females are less stable at the upper trunk post-fatigue than shorter females, particularly in the AP and V directions. As significant results were only found in the female group post-fatigue, the stabilization of the upper trunk during gait appears to be more affected by fatigue in taller females than in shorter females. In the male group, height was only found to be a significant predictor of HarmonicRatio_V_ pre-fatigue at the lower trunk, with taller individuals achieving less smoothness than shorter ones.

A decrease in the smoothness of trunk motion may be the result of a multitude of factors. The finding that the V motion of females is affected at multiple speeds post-fatigue while the V motion of males is not may indicate that the cause of this disturbance relates sex differences in physical and physiological changes to fatiguability experienced during puberty. Taller females were also found to be less stable at the upper trunk in the AP direction at their fast walking speed compared to shorter females, while males showed no such relationship. This may be indicative of decreased stability of the core musculature in taller females compared to shorter females.

There are several limitations in this study that should be specifically addressed in future research. First, this study was limited to individuals, specifically athletes, in the Philadelphia area and must be expanded to include individuals from other geographical and demographic areas to increase the generalizability of the results.

Though necessary for completing 9 min of continuous straight-line walking, walking on a treadmill inherently limits side-to-side motion compared with over-ground walking [26]. This may result in an increase in the dynamic stability of gait measures [26]. The results found here will ultimately need to be translated to an over-ground walking task to better understand how age and height impact gait stability during on-field sports practices in adolescents.

Once the self-reported preferred walking speed and the subsequent calculated slow and fast walking speeds were set, they were not changed during or between walking trials, save the two exceptions to the fast walking speed previously mentioned. Human gait naturally exhibits small-scale stride-to-stride variations in many parameters, including speed. Utilizing a set treadmill speed does not allow for this natural variation in gait to occur. This may force a slightly different gait pattern than over-ground walking [26], in which the participant would be the lone dictator of absolute gait speed stride-to-stride.

Finally, participation in the present study was limited to individuals who regularly and consistently participated in organized sports activities, regardless of sport type. Though sport type was assessed as a covariate, limited sample sizes for each unique sport may have impacted the ability to detect differences between them. Future studies including a larger group from each sport type and each unique sport may help further our understanding of AMA and its sport-specific implications. Additionally, the discovered effects of height and age on the HarmonicRatio may present differently in individuals who do not regularly exercise. This may result from non-athletic individuals exhibiting different patterns of muscle development over this time period or of muscle activation during gait, especially following fatigue, or both. Future research should include non-athletic individuals as well as athletes to improve the generalizability of the findings and to allow for direct comparisons between the groups. The comparison of growth-related impacts on gait stability between athletes and non-athletes will be beneficial in assessing the impacts of sport participation on gait stability during periods of growth. Further studies investigating alterations to the HarmonicRatio during the time period of adolescent physical growth that address these limitations are crucial to enhancing our overall understanding of the phenomenon of adolescent motor awkwardness.

## 6. Conclusions and Future Directions

The objectives of this study were, first, to better understand how one measure of gait stability changes during the adolescent growth phase and, second, to determine how these changes differ between the sexes. Our initial hypothesis that the sexes would differ in how their gait stability changed during the years surrounding peak height growth was supported by our results, though we were surprised to find little effect of height on the HarmonicRatio in males. The findings of this suggest sex differences in the effects of fatigue on gait stability during adolescence. In both sexes, several HarmonicRatio values increased with chronological age. Following a bout of fatiguing exercise, these improvements were eliminated for males and altered for females. The results of this study indicate the need for a reevaluation of sports progression based on chronological age in adolescents.

Overall, the results presented here serve to highlight a notable need for further investigation into the apparently substantial differences between male and female adolescents in the development of gait stability during the years surrounding peak adolescent height growth. The application of this research to youth sports can help guide improvements to the progression of adolescent sports to minimize the risk of injury.

Future studies are necessary to further investigate the effects of absolute height and chronological age on both general gait stability and trunk stability during gait. Additionally, the field of youth sports injury and motor coordination development would benefit from studies investigating the impacts of growth rate, both absolute and relative to height, and of absolute walking speed on gait stability. The results presented here indicate the need for further longitudinal research into the timing of adaptations in motor performance related to age, physical growth, and sex.

## Figures and Tables

**Table 1:** Participants’ physical and gait characteristics by sex.

	Male (*n* = 33)	Female (*n* = 34)
	Mean ± SD (Range)	Mean ± SD (Range)
Physical Characteristics		
Age (years)	13.5 ± 1.78 (10.0–16.7)	12.1 ± 2.11 (8.7–15.9) **
Height (m)	1.66 ± 0.132 (1.33–1.88)	1.57 ± 0.104 (1.39–1.76) **
Gait Characteristics		
Walk-to-Run Transition (m/s)	2.19 ± 0.276 (1.74–2.70)	1.98 ± 0.192 (1.67–2.30) ***
Slow Walking Speed (m/s)	0.965 ± 0.185 (0.49–1.30)	0.923 ± 0.184 (0.70–1.14)
Preferred Walking Speed (m/s)	1.43 ± 0.193 (1.02–1.80)	1.34 ± 0.111 (1.08–1.60) *
Fast Walking Speed (m/s)	1.89 ± 0.201 (1.50–2.30)	1.75 ± 0.151 (1.42–2.10) **

Significant difference between male and female groups indicated with * *p* < 0.05, ** *p* < 0.01, and *** *p* < 0.001.

**Table 2:** Linear regression effects of age on HarmonicRatios in male and female adolescent athletes, adjusted for height (β ± standard error).

	Slow Walking Speed		Preferred Walking Speed		Fast Walking Speed	
	Pre	Post	Pre	Post	Pre	Post
Upper Trunk (C7) Females	β ± Standard Error		β ± Standard Error		β ± Standard Error	
HarmonicRatio_V_	0.112 ± 0.054	0.173 ± 0.049 *	0.160 ± 0.067	0.167 ± 0.046 **	0.163 ± 0.072	0.170 ± 0.068
HarmonicRatio_ML_	0.048 ± 0.069	0.028 ± 0.066	0.139 ± 0.063	0.119 ± 0.071	0.151 ± 0.056	0.127 ± 0.064
HarmonicRatio_AP_	0.158 ± 0.055 *	0.137 ± 0.056	0.249 ± 0.084 *	0.236 ± 0.090	0.275 ± 0.083 *	0.318 ± 0.095 *
Upper Trunk (C7) Males						
HarmonicRatio_V_	0.079 ± 0.049	0.069 ± 0.067	0.107 ± 0.080	0.122 ± 0.081	0.356 ± 0.083 **	0.170 ± 0.068
HarmonicRatio_ML_	0.017 ± 0.084	0.008 ± 0.106	−0.022 ± 0.072	0.018 ± 0.094	0.049 ± 0.077	−0.012 ± 0.078
HarmonicRatio_AP_	0.073 ± 0.050	0.101 ± 0.069	0.094 ± 0.089	0.121 ± 0.086	0.244 ± 0.097 *	0.153 ± 0.109
Lower Trunk (L5) Females						
HarmonicRatio_V_	0.130 ± 0.058	0.195 ± 0.072	0.084 ± 0.072	0.174 ± 0.080 *	0.164 ± 0.071	0.170 ± 0.072
HarmonicRatio_ML_	0.064 ± 0.063	0.054 ± 0.062	0.072 ± 0.093	0.125 ± 0.090	0.118 ± 0.095	0.131 ± 0.112
HarmonicRatio_AP_	0.133 ± 0.080	0.187 ± 0.069	0.134 ± 0.101	0.175 ± 0.096	0.150 ± 0.126	0.320 ± 0.123
Lower Trunk (L5) Males						
HarmonicRatio_V_	0.123 ± 0.068	0.109 ± 0.065	0.031 ± 0.084	0.111 ± 0.099	0.142 ± 0.100	0.132 ± 0.112
HarmonicRatio_ML_	0.079 ± 0.084	−0.027 ± 0.085	0.108 ± 0.127	−0.032 ± 0.085	0.313 ± 0.109 *	0.010 ± 0.103
HarmonicRatio_AP_	0.096 ± 0.095	0.037 ± 0.088	−0.047 ± 0.131	0.017 ± 0.094	0.023 ± 0.144	0.073 ± 0.149

V = vertical, ML = mediolateral, AP = anterior–posterior, slow walking speed = 70% of preferred, preferred walking speed = self-reported preferred walking speed, fast walking speed = 130% of preferred walking speed, pre = pre-fatigue, and post = post-fatigue. All analyses adjusted for height. Significance level indicated with * *p* < 0.01 and ** *p* < 0.001.

**Table 3:** Linear effects of height on HarmonicRatios in female and male adolescent athletes, adjusted for age (β ± standard error).

	Slow Walking Speed		Preferred Walking Speed		Fast Walking Speed	
	Pre	Post	Pre	Post	Pre	Post
Upper Trunk (C7), Females	β ± Standard Error		β ± Standard Error		β ± Standard Error	
HarmonicRatio_V_	−0.051 ± 0.030	−0.098 ± 0.027 *	−0.072 ± 0.037	−0.092 ± 0.025 **	−0.071 ± 0.04	−0.084 ± 0.037
HarmonicRatio_ML_	−0.031 ± 0.041	0.001 ± 0.036	−0.055 ± 0.035	−0.046 ± 0.039	−0.043 ± 0.031	−0.039 ± 0.035
HarmonicRatio_AP_	−0.046 ± 0.030	−0.049 ± 0.031	−0.095 ± 0.046	−0.103 ± 0.049	−0.098 ± 0.046	−0.144 ± 0.052 *
Upper Trunk (C7), Males						
HarmonicRatio_V_	0.022 ± 0.017	0.025 ± 0.021	−0.001 ± 0.028	−0.002 ± 0.026	−0.078 ± 0.029 *	−0.027 ± 0.031
HarmonicRatio_ML_	0.014 ± 0.029	0.030 ± 0.033	0.024 ± 0.025	0.008 ± 0.030	0.005 ± 0.027	0.035 ± 0.025
HarmonicRatio_AP_	0.019 ± 0.017	0.008 ± 0.022	0.015 ± 0.031	−0.021 ± 0.027	−0.041 ± 0.033	0.005 ± 0.034
Lower Trunk (L5), Females						
HarmonicRatio_V_	−0.036 ± 0.032	−0.064 ± 0.040	−0.020 ± 0.040	−0.067 ± 0.044	−0.069 ± 0.040	−0.051 ± 0.039
HarmonicRatio_ML_	−0.003 ± −0.035	−0.008 ± 0.034	−0.009 ± −0.051	−0.054 ± 0.049	−0.045 ± 0.052	−0.048 ± 0.062
HarmonicRatio_AP_	−0.027 ± 0.044	−0.045 ± 0.038	−0.037 ± 0.056	−0.042 ± 0.052	−0.038 ± 0.069	−0.102 ± 0.067
Lower Trunk, (L5) Males						
HarmonicRatio_V_	−0.010 ± 0.023	−0.007 ± 0.021	0.002 ± 0.029	−0.013 ± 0.031	−0.029 ± 0.034	−0.042 ± 0.035
HarmonicRatio_ML_	0.027 ± 0.029	0.034 ± 0.027	0.019 ± 0.044	0.020 ± 0.032	−0.056 ± 0.037	0.000 ± 0.032
HarmonicRatio_AP_	0.031 ± 0.033	0.024 ± 0.028	0.070 ± 0.045	0.016 ± 0.030	0.042 ± 0.050	−0.013 ± 0.047

V = vertical, ML = mediolateral, AP = anterior–posterior, slow walking speed = 70% of preferred, preferred walking speed = self-reported preferred walking speed, fast walking speed = 130% of preferred walking speed, pre = pre-fatigue, post = post-fatigue. All analyses adjusted for age. Significance level indicated with * *p* < 0.01 and ** *p* < 0.001.

## Data Availability

The raw data supporting the conclusions of this article will be made available by the authors on request. The data are not publicly available due to specific ethical and privacy considerations.
